# Association between sleep and ovarian reserve in women undergoing IVF/ICSI: a Mendelian randomization and cross-sectional study

**DOI:** 10.3389/fendo.2026.1970492

**Published:** 2026-09-22

**Authors:** Qunying Fang, Fangfang Li, Zonghui Qiao, Ling Cheng

**Affiliations:** 1 Department of Acupuncture, Shanghai East Hospital, School of Medicine, Tongji University, Shanghai, China; 2 Center for Reproduction and Genetics, Department of Obstetrics and Gynecology, The First Affiliated Hospital of USTC, Division of Life Sciences and Medicine, University of Science and Technology of China, Hefei, China

**Keywords:** diminished ovarian reserve, Mendelian randomization, sleep characteristics, sleep disturbance, sleep duration

## Abstract

**Background:**

Sleep and circadian rhythm disruptions are recognized risk factors for impaired fertility, but their effects on ovarian reserve and underlying mechanisms remain unclear. This study aimed to evaluate the association between sleep characteristics and ovarian reserve in women undergoing *in vitro* fertilization (IVF) and intracytoplasmic sperm injection (ICSI).

**Design:**

A mendelian randomization (MR) analysis of genome-wide association study (GWAS) data assessed the causal link between sleep disorders and FSH levels. A cross-sectional study was then performed to evaluate the association between sleep characteristics and ovarian reserve in women undergoing IVF/ICSI. This observational analysis included data from 1,288 IVF/ICSI patients aged 20–40 years enrolled from June 2023 to May 2024.

**Results:**

MR analysis of GWAS data revealed a significant association between genetically predicted sleep disorders and elevated FSH (Follicle-stimulating hormone) levels (OR = 1.259, P = 0.03). Clinical cross-sectional analyses showed that advanced age (≥35 years, OR: 1.14, 95% CI: 1.07–1.21), unemployment (OR: 0.61, 95% CI: 0.40–0.91), primary infertility (OR: 0.57, 95% CI: 0.35–0.92), sleep disturbance (OR: 2.42, 95% CI: 1.39–4.21), and shorter sleep duration (OR: 1.89, 95% CI: 1.27–2.80) were associated with diminished ovarian reserve (DOR). After adjusting for age, employment status, and infertility type, short sleep duration (6–7 hours and <7 hours) and sleep disturbances remained significantly associated with elevated FSH levels, reduced AMH (Anti-Müllerian hormone) levels, and decreased AFC (antral follicle count). Among women aged <35 years, both short sleep duration and poor sleep quality were significantly associated with DOR. In women aged ≥35 years, only poor sleep quality—not short sleep duration—was significantly associated with DOR.

**Conclusion:**

Shorter sleep duration (<7 hours/night) was associated with DOR in women undergoing IVF/ICSI, particularly in those aged <35 years. In contrast, poor sleep quality adversely affected ovarian reserve regardless of age.

## Introduction

Sleep is an essential physiological process for humans (1). Chronic sleep deficiency has become increasingly prevalent in modern populations due to psychosocial stressors and lifestyle demands. Epidemiological data from the Centers for Disease Control and Prevention (CDC) indicated that 24-48% of adult populations across different U.S. counties experienced short sleep duration (<7 h/night) during 2020 (2). Sleep insufficiency is implicated in the pathogenesis of diverse conditions spanning cardiovascular, neurological, metabolic, and immune systems (3). Previous studies have consistently shown that the optimal amount of sleep for humans is approximately 7 hours (4–7). Sleeping less than 7 hours associated with significantly increased risk of all-cause mortality (8). Systematic reviews suggest that sleep may represent a novel and modifiable factor in reproductive health. Sleep deprivation may exert effects through oxidative stress and inflammatory pathways, which can impair oocyte quality and embryo development (9). To date, most previous studies have focused on a single sleep parameter, and the current evidence remains insufficient to fully elucidate the mechanisms linking sleep patterns to fertility outcomes. Consequently, whether sleep optimization could serve as a therapeutic target in infertility management remains unclear, and further investigations are warranted to clarify these relationships and evaluate potential clinical applications (10).

Optimal ovarian function is fundamental to sustaining both reproductive capacity and overall physiological well-being throughout a woman’s lifespan. Clinically, ovarian reserve is commonly assessed using basal follicle-stimulating hormone (FSH) levels, anti-Müllerian hormone (AMH) concentrations, and antral follicle count (AFC) determined by transvaginal ultrasonography (11). Premature ovarian aging reduces fertility and overall quality of health and leads to premature death (12). Clinical evidence demonstrates that sleep deprivation also adversely affects reproductive function (10, 13). Short sleep duration is associated with diminished conception probability in clinical observations (14). Sleep disturbances are prevalent among *in vitro* fertilization (IVF) patients and persist throughout the entire IVF treatment cycle (15, 16). As IVF progresses, sleep quality may deteriorate—potentially due to the cumulative psychological stress, anxiety related to treatment outcomes, and the physical burden of repeated clinic visits and medication regimens—making routine sleep quality assessment a critical consideration for women undergoing IVF treatment (17).

In 2022, a prospective cohort study based on 1,276 patients found that the association between sleep characteristics (including sleep disturbances, sleep duration, subjective sleep quality, daytime dysfunction, difficulty falling asleep, and night shift experience) and outcomes of IVF/ICSI treatment. The findings revealed that both short sleep duration (<7 hours/night) and sleep disturbance were significantly associated with reduced oocyte yield, lower fertilization rates, and decreased embryo production (18). In 2025, a cross-sectional study including 1,070 participants reported that poor sleep quality is associated with diminished ovarian reserve (DOR) in women of reproductive age (19). Studies have shown that up to 57% of women undergoing IVF/ICSI experience sleep disorders before treatment, with approximately 43% during the stimulation phase and 29% after embryo transfer still reporting sleep problems (20). Given that sleep problems are more common among IVF/ICSI patients than in the general population, and that the proportion of DOR is high in this group (21), the relationship between sleep characteristics and ovarian reserve in women undergoing IVF/ICSI remains poorly understood, particularly due to the lack of a comprehensive sleep feature analysis.

Therefore, a Mendelian randomization (MR) analysis was performed using genome-wide association study (GWAS) data to investigate the potential causal relationship between sleep disorders and follicle-stimulating hormone (FSH) levels. MR analysis is inherently less susceptible to reverse causation. To provide a more comprehensive analysis of the relationship between sleep and ovarian reserve, in the cross-sectional study, we evaluated the associations of sleep characteristics—including sleep disturbances, sleep duration, subjective sleep quality, daytime dysfunction, difficulty falling asleep, and night shift experience—with FSH, anti-Müllerian hormone (AMH), and antral follicle count (AFC) in women undergoing IVF/ICSI. Additionally, we assessed whether maternal age (<35 years vs. ≥35 years) modified the associations between sleep quality/duration and ovarian reserve in this population. We hypothesized that shorter sleep duration and poorer sleep quality are associated with diminished ovarian reserve (DOR) in infertile women undergoing IVF/ICSI.

## Materials and methods

### MR analysis

To infer causality using genetic variants, we performed Mendelian randomization (MR) analysis. Robust instrumental variables were identified from large-scale genome-wide association studies (GWAS). Using the TwoSampleMR package (version 4.3.1) in R, we extracted single nucleotide polymorphisms (SNPs) associated with sleep disorders as exposure variables and examined their effects on FSH. GWAS summary data for sleep disorders were obtained from FinnGen R12 (n = 495,270), and for FSH from IEU OpenGWAS (prot-a-528, n = 3,301). After LD clumping (R² < 0.001) and harmonization, 123 SNPs (P < 5×10^−8^) were selected as instrumental variables. The inverse-variance weighted (IVW) method was applied to assess the association between sleep disorders and FSH. Sensitivity analysis was carried out using leave-one-out validation.

### Study design

This cross-sectional study included 1,288 female patients aged 20–40 years who visited the Center for Reproductive Genetics at the First Hospital of the University of Science and Technology of China between June 2023 and May 2024. On the day of their visit, participants completed questionnaires on lifestyle, demographic characteristics, and the Pittsburgh Sleep Quality Index (PSQI) (22). Exclusion criteria included: received endocrine medications within the last 3 months, incomplete questionnaires, polycystic ovary syndrome (PCOS), endometriosis and other ovarian disease, history of ovarian surgery. The diagnoses of PCOS, endometriosis, and other ovarian disorders were retrieved from physicians’ formal diagnostic records in the electronic medical record (EMR) system. The patient selection process is detailed in **Figure 1**.

**Figure 1:**
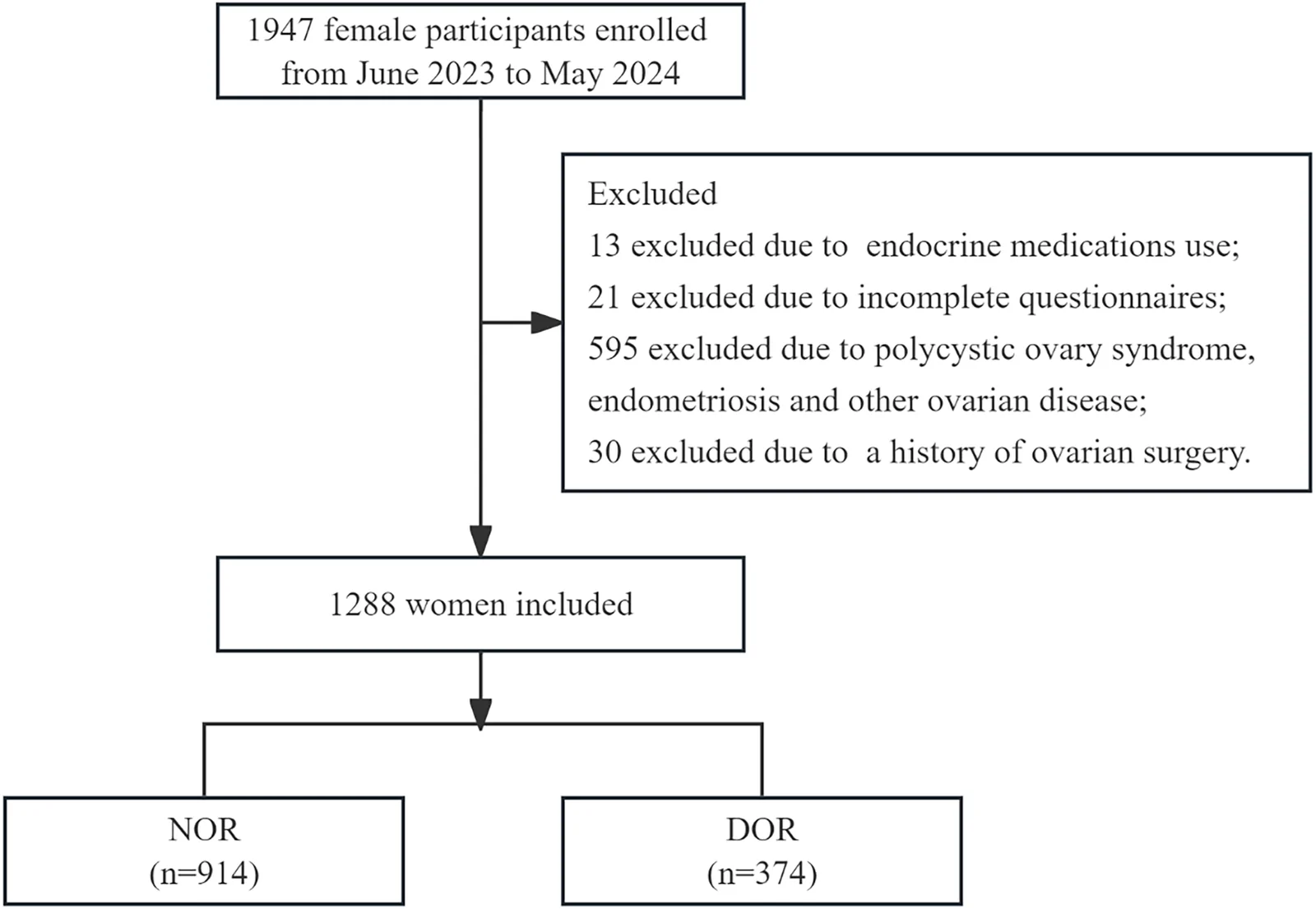
Flow chart of the participant selection process.

### Ethics approval and consent to participate

This study was conducted in accordance with the Declaration of Helsinki and approved by the Ethics Committee of the First Affiliated Hospital of the USTC (ethical approval No. XJS2022-1-13(YJ); approval date: 30 June 2022). All participants gave their consent to participate prior to the inclusion in the study.

### Assessment of sleep characteristics

The PSQI comprises seven components (subjective sleep quality, sleep latency, sleep duration, habitual sleep efficiency, sleep disorders, sleep medication use, and daytime dysfunction), with total scores ranging from 0 to 21. A score of ≤5 indicates normal sleep quality, whereas >5 indicates poor sleep quality. We assessed sleep duration (≥7 h, 6-7h, <6h), subjective sleep quality (Good or Poor), trouble falling asleep (never, less than once a week, once or twice a week or three times or more a week), sleep disturbance (Yes or No), daytime dysfunction (Yes or No), and night shift experience (Yes or No).

### Covariates

Standardized questionnaires were used to collect comprehensive baseline data on demographic characteristics (age, education level, occupation, and body mass index [BMI]), lifestyle factors (smoking status, alcohol consumption frequency, exercise habits, and sleep characteristics including disturbances, duration, subjective quality, daytime dysfunction, difficulty falling asleep, and night shift experience), and detailed medical history (infertility type [primary/secondary], infertility duration, number of prior miscarriages, history of dysmenorrhea, previous tubal surgery, chronic disease history, and neurological or psychiatric disorders) (23). Infertility type was classified according to the World Health Organization definition: primary infertility was defined as never having conceived, and secondary infertility as having at least one prior pregnancy but currently being unable to conceive again.

### Assessment of ovarian reserve

To evaluate ovarian reserve, we obtained day 3 FSH and AMH levels from medical records (measured by chemiluminescent immunoassay) and performed transvaginal ultrasound to determine AFC in all participants (11). Reproductive hormones were quantified using chemiluminescence immunoassay (Roche Cobas e 801, Basel, Switzerland), and AMH was measured via electrochemiluminescence immunoassay (Abbott Alinity i, Abbott Park, IL, USA). AFC was defined as the total number of follicles sized 2–9 mm in both ovaries during the early follicular phase. To ensure consistency, all ultrasound assessments were performed by the same experienced gynecologist under transvaginal guidance (19). DOR was defined as meeting at least one of the following criteria: AFC <7, AMH <1.1 ng/mL, or day 3 FSH ≥10 IU/L (24, 25).

### Statistical analysis

Continuous variables are shown as mean ± SD, with group comparisons performed via independent-samples t-tests. Categorical data are expressed as n (%) and evaluated using χ² or Fisher’s exact tests. Univariate logistic regression was applied to explore links between ovarian reserve parameters and Baseline characteristics. Statistical significance was set at two-tailed P < 0.05. Age-stratified analyses (<35 vs. ≥35 years) assessed potential effect modification by sleep duration and quality, the latter dichotomized by PSQI score (≤5 vs. >5). SPSS 26.0 (IBM Corp) was used for all analyses, with two-tailed testing and α = 0.05.

## Results

### Genetic evidence for a causal link between sleep disorders and FSH levels

To examine whether sleep disorders causally affect FSH, we carried out MR using GWAS summary data. Four different MR techniques (IVW, MR-Egger, Weighted Median, and Weighted Mode) consistently identified sleep problems as raising FSH levels (**Figure 2A**). We selected 116 genetic variants related to sleep troubles as instrumental variables. A funnel plot showed a highly symmetric spread of SNP effect estimates, implying negligible directional pleiotropy and confirming analysis sturdiness (**Figure 2B**). With the inverse-variance weighted (IVW) method, genetically proxied sleep disturbances exhibited a meaningful link to higher FSH (OR = 1.259, P = 0.03; **Figure 2C**). To further verify result stability, we performed leave-one-out sensitivity testing by excluding each SNP one after another. The aggregated estimates stayed unchanged; confidence intervals varied little and always remained on one side of the null after any single SNP was removed (**Figure 2D**). This pattern suggests no individual SNP overly affected the total relationship. In sum, this MR delivers genetic proof backing a causative connection from frequent sleep issues to elevated FSH levels in women.

**Figure 2:**
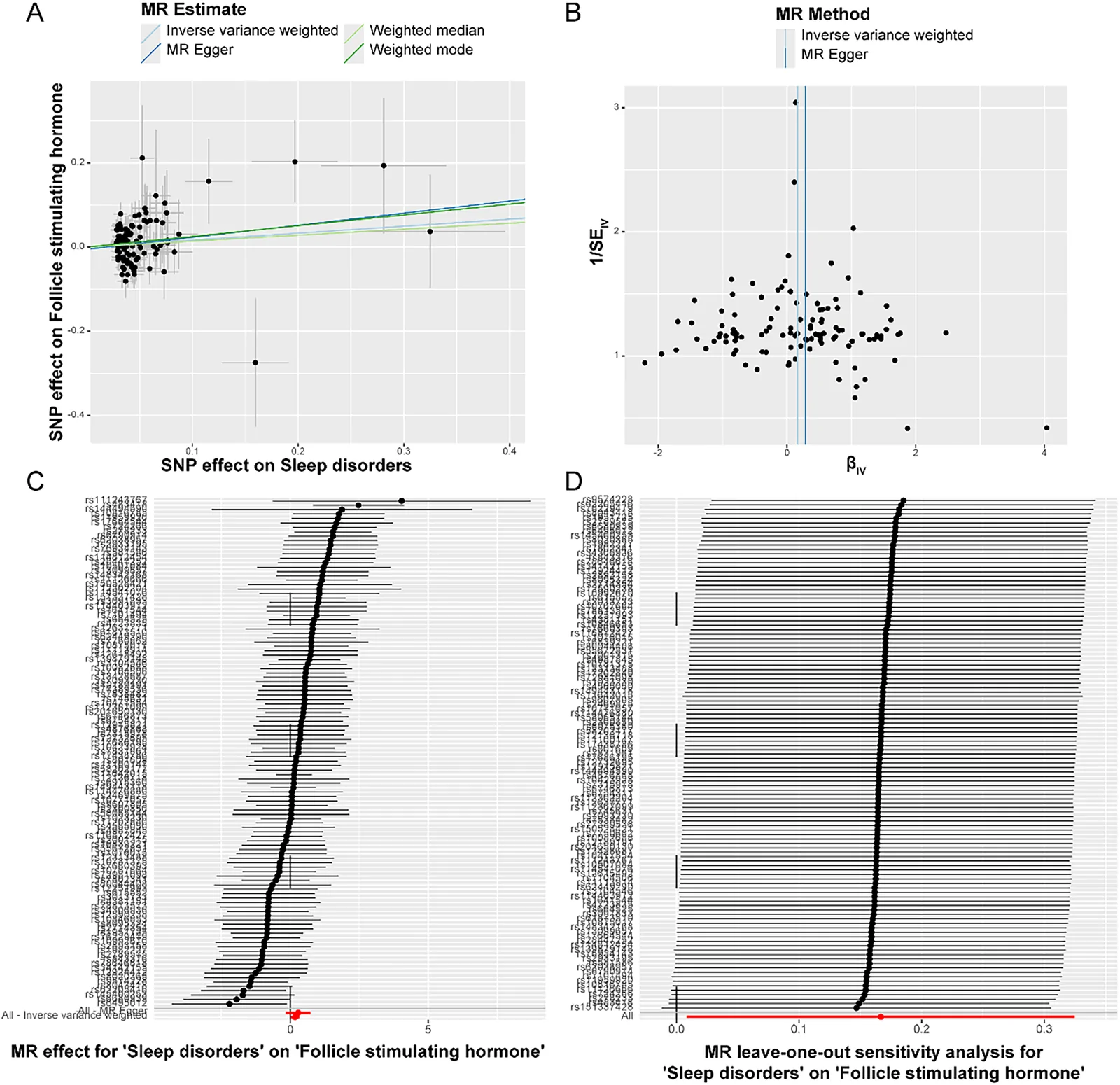
Mendelian randomization analysis of sleep disorders with FSH levels. **(A)** The scatter plot of the causal relationship between sleep disorders and FSH levels is based on four different methods: inverse variance weighted, MR Egger, weighted median, and weighted mode. **(B)** The funnel plot displays the overall heterogeneity of the Mendelian randomization estimates for the effect of sleep disorders on FSH. **(C)** The forest plot shows the causal relationship between each SNP and FSH levels. **(D)** The forest plot presents the leave-one-out method for sensitivity analysis. MR, Mendelian randomization.

### Baseline characteristics

**Table 1** shows the baseline characteristics of the 1288 patients. The mean age of all participants was 32.35 ± 4.13 years. Women with DOR were significantly older than those with normal ovarian reserve (NOR) (31.64 ± 4.05 vs 34.09 ± 4.02, *P* = 0.000). BMI and education were comparable between the two groups of women. Women with NOR significantly higher employment rates compared to those women with DOR (82.39% vs 75.67%, *P* = 0.006). The majority of patients had no history of smoking (98.60%) or alcohol consumption (94.72%), and the overall mean duration of infertility was 2.48 ± 2.12 years. These characteristics showed no statistically significant differences between the two groups(*P*>0.05). Most of the patients did not experience salpingectomy (92.55%), dysmenorrhea (83.38%), and fewer than 3 prior induced abortions (94.95%). Patients with DOR had predominantly primary infertility, which was statistically different between the two groups (59.63 vs 50.33, *P* = 0.002). These characteristics showed no statistically significant differences between the two groups(*P*>0.05). DOR patients had significantly reduced nocturnal sleep time versus NOR patients(*P* = 0.000). The DOR group had significantly more frequent poor subjective sleep quality than the NOR group (22.73% vs 11.93%, *P* = 0.009). The correlation with sleep disturbances was significantly more pronounced in the DOR group compared to the NOR group (38.77% vs 27.68%, *P* = 0.000). No significant differences were observed between the two groups in terms of trouble falling asleep, daytime dysfunction, or night shift experience(*P*>0.05).

**Table 1:** Baseline characteristics of all participants (n=1288).

Characteristics	Total (n=1288)	NOR (n=914)	DOR (n=374)	P-value
Age (years)	32.35 ± 4.13	31.64 ± 4.05	34.09 ± 4.02	0.000
BMI (kg/m^2^)	22.91 ± 3.23	22.77 ± 3.31	23.24 ± 2.99	0.223
Educational level, n (%)				0.399
Middle school and below	435 (33.78)	310 (33.92)	125 (33.42)	
High school	445 (34.55)	324 (35.45)	121 (32.35)	
College and above	408 (31.68)	280 (30.63)	128 (34.22)	
Employed, n (%)				0.006
Yes	1036 (80.43)	753 (82.39)	283 (75.67)	
No	252 (19.57)	161 (17.61)	91 (24.33)	
Smoking, n (%)				0.354
Never	18 (1.40)	11 (1.20)	7 (1.87)	
Ever	1270 (98.60)	903 (98.80)	367 (98.13)	
Alcohol drinking, n (%)				0.149
Yes	68 (5.28)	43 (4.70)	25 (6.68)	
No	1220 (94.72)	871 (95.30)	349 (93.32)	
Hypnotic medications, n (%)				0.423
Yes	31 (2.41)	24 (2.63)	7 (1.87)	
No	1257 (97.59)	890 (97.37)	367 (98.13)	
Duration of infertility (years)	2.48 ± 2.12	2.46 ± 1.98	2.52 ± 2.21	0.735
Salpingectomy history, n (%)				0.335
Yes	96 (7.45)	64 (7.00)	32 (8.56)	
No	1192 (92.55)	850 (93.00)	342 (91.44)	
Dysmenorrhea, n (%)				0.311
Yes	214 (16.62)	158 (17.29)	56 (14.97)	
No	1074 (83.38)	756 (82.71)	318 (85.03)	
Induced abortion, n (%)				0.752
< 3	1223 (94.95)	869 (95.08)	354 (94.65)	
≥3	65 (5.05)	45 (4.92)	20 (5.35)	
Infertility, n (%)				0.002
Primary infertility	683 (53.03)	460 (50.33)	223 (59.63)	
Secondary infertility	605 (46.97)	454 (49.67)	151 (40.37)	
Exercise (times/week)				0.654
0	109 (8.46)	73 (7.99)	36 (9.63)	
1	405 (31.44)	285 (31.18)	120 (32.09)	
2	573 (44.49)	408 (44.64)	165 (44.12)	
3	201 (15.61)	148 (16.19)	53 (14.17)	
Nocturnal sleep duration (h)				0.000
≥7h	760 (59.0)	594 (64.98)	166 (44.39)	
6-7h	443 (34.39)	280 (30.63)	163 (43.58)	
<6h	85 (6.60)	40 (4.38)	45 (12.03)	
Subjective sleep quality				0.009
Good	1099 (85.33)	795 (88.07)	304 (77.27)	
Poor	189 (14.67)	119 (11.93)	70 (22.73)	
Trouble falling asleep (times/week)				0.072
0	532 (41.30)	383 (41.90)	149 (39.84)	
1	312 (24.22)	230 (25.16)	82 (21.93)	
2	299 (23.21)	211 (23.09)	88 (23.53)	
3	145 (11.26)	90 (9.85)	55 (14.70)	
Sleep disturbance				0.000
No	889 (69.02)	661 (72.32)	229 (61.23)	
Yes	399 (30.98)	253 (27.68)	145 (38.77)	
Daytime dysfunction				0.470
No	924 (71.74)	661 (73.32)	263 (70.32)	
Yes	364 (28.26)	253 (27.68)	111 (29.68)	
Night shift experience				0.712
No	1170 (90.84)	832 (91.03)	338 (90.37)	
Yes	118 (9.16)	82 (8.97)	36 (9.63)	
Assessment of ovarian reserve				
FSH (IU/L)	7.78 ± 4.45	6.41 ± 2.36	11.12 ± 3.34	0.000
E2 (pg/ml)	40.10 ± 11.86	36.52 ± 11.56	48.86 ± 12.42	0.000
AMH (ng/ml)	3.16 ± 1.83	3.92 ± 1.34	1.30 ± 0.56	0.000
AFC (n)	10.60 ± 6.10	12.97 ± 4.32	4.80 ± 3.24	0.000

Data presented as mean (SD) or number (%) as appropriate. BMI, body mass index; FSH, follicle-stimulating hormone; E2, Estradiol**;** AMH, Anti-Müllerian Hormone; AFC, antral follicle count.

### Univariate logistic regression analysis

According to the definition of DOR, logistic regression analysis was performed with AFC <7 or AMH <1.1 ng/mL or FSH ≥10 IU/L as the dependent variables (**Table 2**), and the treatment of the variables is shown in **Supplementary Table 1**. Advanced age was significantly correlated with DOR (OR:1.14, 95% CI:1.07-1.21). Unemployment status was significant association with DOR (OR:0.61, 95% CI:0.40-0.91). Women with primary infertility demonstrated a significantly stronger association with DOR compared to secondary infertility (OR:0.57, 95% CI:0.35-0.92). Short sleep duration (both 6–7 h and <6 h) showed a significant association with DOR. Sleep disturbance demonstrated a significant correlation with DOR (OR:2.42, 95%CI:1.39-4.21).

**Table 2:** Univariable logistic regression results for the association between the risk factors with diminished ovarian reserve.

Variables						95% CI	
	B	SE	Z	*P*-value	OR	Lower	Upper
Age	0.126	0.031	16.739	0.000	1.14	1.07	1.21
BMI	0.20	0.028	0.547	0.459	1.02	0.97	1.08
Educational level	0.253	0.405	0.391	0.532	1.29	0.58	2.85
Employed	-0.503	0.206	5.943	0.015	0.61	0.40	0.91
Smoking	-0.04	0.044	0.817	0.366	0.96	0.88	1.05
Alcohol drinking	-0.357	0.324	1.212	0.271	0.70	0.37	1.32
Hypnotic medications	0.464	0.518	0.802	0.371	1.59	0.58	4.39
Duration of infertility	0.017	0.041	0.174	0.677	1.02	0.94	1.10
Salpingectomy history	0.378	0.34	1.238	0.266	1.46	0.75	2.84
Dysmenorrhea	-0.154	0.364	0.180	0.671	0.86	0.42	1.75
Induced abortion	-0.215	0.390	0.303	0.582	0.81	0.38	1.73
Infertility	-0.564	0.246	5.241	0.022	0.57	0.35	0.92
Exercise			1.989	0.575			
1	-0.186	0.330	0.318	0.573	0.83	0.43	1.59
2	-0.357	0.324	1.212	0.271	0.70	0.37	1.32
3	-0.435	0.375	1.349	0.245	0.65	0.31	1.35
Nocturnal sleep duration (h)							
6-7h	0.495	0.198	6.270	0.012	1.64	1.11	2.42
<6h	0.628	0.378	2.764	0.020	1.88	0.89	3.93
Subjective sleep quality	0.441	0.679	0.422	0.516	1.55	0.41	5.88
Trouble falling asleep (times/week)							
1	-0.227	0.217	1.098	0.295	0.79	0.49	1.25
2	-0.157	0.245	0.413	0.520	0.78	0.47	1.31
3	-0.188	0.338	0.309	0.578	0.69	0.69	1.36
Sleep disturbance	0.882	0.283	9.715	0.002	2.42	1.39	4.21
Daytime dysfunction	0.220	0.237	0.862	0.353	1.25	0.78	1.98
Night shift experience	0.317	0.363	0.764	0.382	1.37	0.67	2.80

B, regression coefﬁcient; SE, Standard Error; BMI, body mass index; CI, conﬁdence interval; OR, odds ratio; Z, Z-value or Z-score. FSH, follicle-stimulating hormone; AMH, Anti-Müllerian Hormone; AFC, antral follicle count.

### Multivariate regression analysis

Multivariate regression analysis incorporated all variables with *P* < 0.05 in preliminary analyses, as presented in **Table 2**. It is well-established in reproductive endocrinology that BMI is associated with both sleep quality and ovarian reserve. Sleep duration, subjective sleep quality, trouble falling asleep, sleep disturbance, daytime dysfunction, and night shift experience were the independent variable, adjusted for BMI, age, employed and type of infertility. Results showed that a sleep duration of 6–7 hours was associated with elevated FSH (adjusted odds ratio [AOR]: 2.12; 95% CI, 1.24-3.73), reduced AMH (AOR:1.11; 95%CI, 0.32-4.27), and decreased AFC (AOR:1.43, 95%CI, 1.02-2.19). A sleep duration of <6 hours was associated with elevated FSH (AOR: 2.85; 95% CI, 1.03-8.37), and decreased AFC (AOR:2.21, 95%CI, 1.13-4.86). Sleep disturbance was associated with elevated FSH (AOR: 3.87; 95% CI, 1.63-9.13), reduced AMH (AOR:2.43; 95%CI, 1.15-5.56), and decreased AFC (AOR:1.92, 95%CI,1.18-3.27). FSH, AMH and AFC overall outcomes were also significantly associated with shorter sleep duration (6–7 hours and <7 hours), and sleep disturbance. Trouble falling asleep (3 times/week) was significantly associated with reduced AMH levels (AOR = 1.54; 95% CI: 0.56–4.39) (**Table 3**).

**Table 3:** Multivariate regression was used to analyze the association between sleep characteristics and diminished ovarian reserve.

Characteristics	FSH≥10	AMH<1.1	AFC<7	AMH <1.1 OR AFC <7 OR FSH≥10
Nocturnal sleep duration (h)				
≥7h	Ref	Ref	Ref	Ref
6-7h	**2.12(1.24,3.73)**	**1.11(0.32,4.27)**	**1.43(1.02,2.19)**	**1.63(1.14,2.38)**
<6h	**2.85(1.03,8.37)**	2.24(1.27,3.98)	**2.21(1.13,4.86)**	**1.86(0.91,3.92)**
Subjective sleep quality				
Good	Ref	Ref	Ref	Ref
Poor	1.15(0.58,2.41)	1.83(0.76,4.60)	1.17(0.29,4.85)	1.54(0.43,5.87)
Trouble falling asleep (times/week)				
0	Ref	Ref	Ref	Ref
1	0.60(0.32,1.05)	1.07(0.58,2.15)	0.86(0.57,1.35)	0.77(0.51,1.23)
2	0.62(0.31,0.40)	0.98(0.43,2.18)	0.97(0.59,1.63)	0.76(0.49,1.30)
3	0.65(0.21,2.29)	**1.54(0.56,4.39)**	0.84(0.44,1.73)	0.65(0.37,1.32)
Sleep disturbance				
No	Ref	Ref	Ref	Ref
Yes	**3.87(1.63,9.13)**	**2.43(1.15,5.56)**	**1.92(1.18,3.27)**	**2.41(1.42,4.08)**
Daytime dysfunction				
No	Ref	Ref	Ref	Ref
Yes	0.54(0.37,1.14)	1.22(0.68,2.32)	1.23(0.87,1.91)	1.25(0.88,1.96)
Night shift experience				
No	Ref	Ref	Ref	Ref
Yes	0.64(0.28,2.43)	0.82(0.40,2.25)	1.56(0.85,3.12)	1.32(0.73,2.54)

Models were adjusted for BMI, age, employment status, and type of infertility. Bold values indicate statisticalsignificance. Ref, reference group.

### Effect of age on sleep quality and ovarian reserve

Age is an independent risk factor for ovarian reserve function. To further investigate whether age modulates the association between sleep quality and ovarian reserve, we stratified patients into two age groups (<35 years and ≥35 years). Using sleep quality as the dependent variable while adjusting for BMI, employment status (employed/unemployed) and infertility type (primary/secondary), our analysis revealed that in the <35-year subgroup, poor sleep quality was significantly associated with an elevated prevalence of DOR compared to those with normal sleep quality. The adjusted odds ratios were as follows: FSH (AOR 3.47, 95% CI 1.89-6.39), AMH (AOR 2.47, 95% CI 1.21-5.06), AFC (AOR 1.75, 95% CI 1.23-2.74), and composite AMH/AFC/FSH (AOR 2.02, 95% CI 1.36-3.25). Similarly, in the ≥35-year subgroup, PSQI scores >5 showed significant association with DOR. Notably, the age-stratified analysis revealed no significant interactive effect of age and sleep quality scores on DOR (**Table 4**).

**Table 4:** Multivariate regression was used to analyze the association between sleep quality and DOR grouped by age.

Variables	Age<35y (n=894)		Age ≥35y (n=394)		*P* for interaction
	PSQI ≤ 5 (n=572)	PSQI≥5 (n=322)	PSQI ≤ 5 (n=317)	PSQI≥5 (n=77)	
FSH≥10					0.128
n (%)	59 (10.31)	82 (25.47)	67 (21.14)	17 (36.36)	
OR (95% CI)	1 (reference)	3.89 (2.19-6.93)	1 (reference)	5.75 (2.48-13.29)	
AOR (95% CI)	1 (reference)	3.47 (1.89-6.39)	1 (reference)	6.59 (2.88-16.45)	
AMH<1.1					
n (%)	78 (13.64)	77 (23.91)	88 (27.76)	38 (49.35)	0.246
OR (95% CI)	1 (reference)	3.47 (1.74-6.91)	1 (reference)	3.09 (1.18-8.05)	
AOR (95% CI)	1 (reference)	2.47 (1.21-5.06)	1 (reference)	4.21 (1.45-11.29)	
AFC<7					
n (%)	75 (13.11)	89 (27.64)	63 (19.87)	33 (42.86)	0.067
OR (95% CI)	1 (reference)	2.95 (1.61-3.88)	1 (reference)	2.94 (1.497-5.79)	
AOR (95% CI)	1 (reference)	1.75 (1.23-2.74)	1 (reference)	2.86 (1.43-5.74)	
AMH <1.1 OR AFC <7 OR FSH≥10					
n (%)	86 (15.03)	106 (32.92)	138 (43.53)	29(61.04)	0.110
OR (95% CI)	1 (reference)	2.76 (1.82-4.19)	1 (reference)	2.20 (1.15-4.23)	
AOR (95% CI)	1 (reference)	2.02 (1.36-3.25)	1 (reference)	2.61 (1.32-5.23)	

Data presented as number (%). FSH, follicle-stimulating hormone; AMH, Anti-Müllerian Hormone; AFC, antral follicle count; AOR, adjusted odds ratio. For the <35 years subgroup: n = 894, mean age 30.23 ± 3.82 years. For the ≥35 years subgroup: n = 394, mean age 37.16 ± 4.87 years.

### Effect of age on sleep duration and ovarian reserve

We further examined whether age modifies the association between sleep duration and ovarian reserve. Patients were categorized by age as <35 and ≥35 years. Sleep duration was used as the independent variable and adjusted for BMI, employment status (employed/unemployed) and infertility type (primary/secondary). In the <35-year-old subgroup, women with short sleep duration (<7 h/night) demonstrated a significantly associated with DOR. AORs were as follows: FSH (AOR, 2.19; 95% CI, 1.41-3.76), AMH (AOR, 3.75; 95% CI, 2.24-6.52), AFC (AOR, 2.41; 95% CI, 1.65-3.74), FSH (AOR, 2.31; 95% CI, 1.35-3.94), and AMH or AFC or FSH (AOR, 2.52; 95% CI, 1.69-3.64). However, among women aged ≥35 years, no significant difference was observed between the short (<7 h) and adequate (≥7 h) sleep duration groups. Notably, the age-stratified analysis revealed significant interactive effect of age and sleep duration scores on DOR (**Table 5**).

**Table 5:** Multivariate regression was used to analyze the association between sleep duration and DOR grouped by age.

Variables	Age<35y (n=894)		Age ≥35y (n=394)		*P* for interaction
	≥7h(n=569)	<7h(n=325)	≥7h (n=191)	<7h (n=203)	
FSH≥10					0.000
n (%)	63 (11.07)	54 (16.62)	35(18.32)	39 (19.21)	
OR (95% CI)	1 (reference)	2.193 (1.29-3.73)	1 (reference)	1.41 (0.74-2.68)	
AOR (95% CI)	1 (reference)	2.19 (1.41-3.76)	1 (reference)	1.32 (0.81-2.57)	
AMH<1.1					0.002
n (%)	70 (12.30)	84 (25.85)	72 (37.70)	49 (39.41)	
OR (95% CI)	1 (reference)	3.14 (1.81-5.45)	1 (reference)	1.61 (0.76-3.40)	
AOR (95% CI)	1 (reference)	3.75 (2.24-6.52)	1 (reference)	1.58 (0.78-3.50)	
AFC<7					0.001
n (%)	130 (22.85)	103 (31.69)	67 (35.08)	72 (35.47)	
OR (95% CI)	1 (reference)	2.45 (1.67-3.60)	1 (reference)	1.14 (0.69-1.90)	
AOR (95% CI)	1 (reference)	2.41 (1.69-3.64)	1 (reference)	1.21 (0.75-1.98)	
AMH <1.1 OR AFC <7 OR FSH≥10					0.001
n (%)	150 (26.36)	116 (35.69)	78 (40.83)	88 (43.34)	
OR (95% CI)	1 (reference)	2.44 (1.67-3.58)	1 (reference)	1.12 (0.68-1.87)	
AOR (95% CI)	1 (reference)	2.51 (1.69-3.62)	1 (reference)	1.15 (0.78-1.92)	

Data presented as number (%). FSH = follicle-stimulating hormone; AMH, Anti-Müllerian Hormone; AFC, antral follicle count; AOR, adjusted odds ratio. For the <35 years subgroup: n = 894, mean age 30.23 ± 3.82 years. For the ≥35 years subgroup: n = 394, mean age 37.16 ± 4.87 years.

## Discussion

Our study demonstrated that both short sleep duration (<7 hours/night) and sleep disturbance are associated with diminished ovarian reserve in women undergoing IVF/ICSI. Notably, short sleep duration exerts a stronger adverse effect in women aged <35 years, whereas poor sleep quality significantly impairs ovarian reserve regardless of age.

First, the genetic evidence provided by MR analysis indicates that sleep disorders may contribute to an increased risk of elevated FSH levels. It should be noted that, although the MR analysis provides genetic-level causal evidence linking sleep disorders to elevated FSH levels, it does not directly support a causal association between short sleep duration and altered FSH levels. Accordingly, the MR findings of this study should not be overextended to the context of sleep duration, and caution is required when generalizing the causal inferences obtained here.

In this study, 40.99% of participants reported short sleep duration (<7h/night). Epidemiological evidence has established significant associations between sleep deprivation and various reproductive health disturbances in the general population, including menstrual irregularities (26), premenstrual syndrome (27), dysregulation of reproductive hormone profiles (28), and prolonged gestational duration (14). This cross-sectional study analysis examined potential correlations between clinical characteristics and lifestyle factors with ovarian reserve parameters in women undergoing IVF/ICSI. Logistic regression analysis revealed that advanced age, unemployment, primary infertility, sleep disturbances, and short sleep duration (6–7 hours and <7 hours) were significantly associated with DOR. Advanced age is consistently recognized as a key independent predictor of DOR, with ovarian reserve exhibiting a progressive age-related decline (29–31). Accumulating evidence also supports psychological stress as a significant modulator of ovarian reserve (32, 33). Of note, unemployed women exhibited a significantly higher risk of DOR compared with their employed counterparts, potentially attributable to greater exposure to socioeconomic stressors in this population (34–36).

In this study, 30.98% of participants exhibited sleep disturbances, a prevalence comparable to the 29.35% reported in previous studies (19). Sleep disturbances are prevalent among IVF patients and persist throughout the treatment cycle (15, 16). Routine sleep quality assessment a critical consideration for women undergoing IVF treatment (17). Study has shown that women with DOR had nearly three times the likelihood of reporting sleep disturbances compared with those with other causes of infertility (37). However, the causal relationship between sleep and DOR remains unclear in IVF/ICSI populations, with a notable lack of systematic investigations to date. Our study demonstrates a significant association between sleep disturbances and DOR in IVF/ICSI patients, which aligns with Lin et al.’s findings of similar correlations in the general reproductive-aged population (19).

Sleep may impair fertility by disrupting the hypothalamic-pituitary-ovarian (HPO) axis and inducing endocrine dysfunction. Overloaded work schedules, sleep deprivation, poor sleep quality, and excessive mobile phone use are pervasive in modern society and can inevitably disrupt circadian rhythms (10, 38). Circadian disruption, in turn, may impair ovarian function through alterations in the HPO axis (39). A longitudinal study of 365 midlife women during menopausal transition found that both longer sleep duration (measured by home polysomnography) and poorer self-reported sleep quality were associated with accelerated FSH rate of change. Additionally, estradiol levels exhibited an inverse relationship with sleep quality, suggesting that sleep patterns influence characteristic hormonal changes during the menopausal transition (40). In addition, a prospective study of 259 eumenorrheic women demonstrated that each additional hour of daily sleep was associated with a significant 3.9% increase in mean estradiol concentration and a 9.4% increase in luteal phase progesterone levels (28).

Our further stratified analysis revealed a statistically significant interaction between age with sleep duration and ovarian reserve in the age-based subgroup analyses, suggesting that the association between sleep duration and ovarian reserve was not consistent across age. In patients <35 years old, shorter sleep duration showed significant associations with DOR, while no such association was observed in women ≥35 years of age. Accumulating evidence identifies chronic sleep quality impairment as a significant independent risk factor for DOR in women aged 36–40 years (41). Further analysis revealed a consistent association between sleep quality and ovarian reserve function that remained stable across different age groups, with no significant effect modification by age. These results are consistent with previous reports by Li et al (19). Sufficient sleep duration requirements vary across the lifespan and from person to person (4). Since sleep requirements decrease with advancing age, we hypothesize that advanced-age females (>35 years) may show diminished sensitivity to sleep duration variations. We will further investigate the underlying mechanisms using animal models.

There are several limitations. 1) All sleep measurements in this study rely exclusively on self-report data collected via the PSQI questionnaire, with no validation from objective sleep monitoring approaches (e.g., polysomnography or consumer wearable devices). 2) The cross-sectional study design precludes the establishment of temporal sequences between exposure and outcome, meaning reverse causality cannot be ruled out. 3) The study population is composed entirely of infertile women receiving IVF/ICSI treatment, so extrapolation of the study findings to the general population of fertile women should be conducted with caution. 4) Given that psychological distress is a well-established confounder that influences both subjective sleep quality and neuroendocrine regulation of the ovarian reserve, the absence of these indicators may introduce unmeasured confounding bias. Future prospective cohort studies are warranted to adjust for these psychological covariates. 5) Given that our study population was limited to infertile women aged 20–40 years undergoing IVF/ICSI, future studies should include a broader age range of reproductive-aged women (e.g., 18–50 years, covering both naturally conceiving and perimenopausal populations) to further validate the generalizability of our findings and to better characterize the age-dependent associations between sleep and ovarian reserve.

## Conclusion

Shorter sleep duration(<7h) is associated with DOR in women undergoing IVF/ICSI, particularly in those under 35 years old. In contrast, poor sleep quality negatively impacts ovarian reserve regardless of age. These findings highlight sleep characteristics as potentially modifiable factors for ovarian reserve, particularly in younger reproductive-aged women.

## Data Availability

The original contributions presented in the study are included in the article/**Supplementary Material**. Further inquiries can be directed to the corresponding author.

## Supplementary material

Supplementary Table 1 Variable assignment.

## References

[1] Grandner MA Fernandez FX . The translational neuroscience of sleep: a contextual framework. Science. (2021) 374:568–73. doi: 10.1126/science.abj8188 34709899 PMC8761057

[2] CDC . Short Sleep Duration Among Us Adults (2020). Available online at: https://wwwcdcgov/sleep/data-research/facts-stats/adults-sleep-facts-and-statshtml (Accessed May 15, 2024).

[3] Garbarino S Lanteri P Bragazzi NL Magnavita N Scoditti E . Role of sleep deprivation in immune-related disease risk and outcomes. Commun Biol. (2021) 4:1304. doi: 10.1038/s42003-021-02825-4 34795404 PMC8602722

[4] Hirshkowitz M Whiton K Albert SM Alessi C Bruni O DonCarlos L . National Sleep Foundation’s sleep time duration recommendations: methodology and results summary. Sleep Health. (2015) 1:40–3. doi: 10.1016/j.sleh.2014.12.010 29073412

[5] Chen Y Tan F Wei L Li X Lyu Z Feng X . Sleep duration and the risk of cancer: a systematic review and meta-analysis including dose-response relationship. BMC Cancer. (2018) 18:1149. doi: 10.1186/s12885-018-5025-y 30463535 PMC6249821

[6] Li Y Sahakian BJ Kang J Langley C Zhang W Xie C . The brain structure and genetic mechanisms underlying the nonlinear association between sleep duration, cognition and mental health. Nat Aging. (2022) 2:425–37. doi: 10.1038/s43587-022-00210-2 37118065

[7] Chaput J-P Dutil C Featherstone R Ross R Giangregorio L Saunders TJ . Sleep duration and health in adults: an overview of systematic reviews. Appl Physiol Nutr Metab. (2020) 45:S218–31. doi: 10.1139/apnm-2020-0034 33054337

[8] Liu TZ Xu C Rota M Cai H Zhang C Shi MJ . Sleep duration and risk of all-cause mortality: a flexible, non-linear, meta-regression of 40 prospective cohort studies. Sleep Med Rev. (2017) 32:28–36. doi: 10.1016/j.smrv.2016.02.005 27067616

[9] Li J Huang Y Xu S Wang Y . Sleep disturbances and female infertility: a systematic review. BMC Women's Health. (2024) 24:643. doi: 10.1186/s12905-024-03508-y 39707272 PMC11660991

[10] Caetano G Bozinovic I Dupont C Léger D Lévy R Sermondade N . Impact of sleep on female and male reproductive functions: a systematic review. Fertil Steril. (2021) 115:715–31. doi: 10.1016/j.fertnstert.2020.08.1429 33054981

[11] Medicine PCotASfR . Testing and interpreting measures of ovarian reserve: a committee opinion. Fertil Steril. (2020) 114:1151–7. doi: 10.1016/j.fertnstert.2020.09.134 33280722

[12] Muka T Oliver-Williams C Kunutsor S Laven JS Fauser BC Chowdhury R . Association of age at onset of menopause and time since onset of menopause with cardiovascular outcomes, intermediate vascular traits, and all-cause mortality: a systematic review and meta-analysis. JAMA Cardiol. (2016) 1:767–76. doi: 10.1001/jamacardio.2016.2415 27627190

[13] Eisenberg E Legro RS Diamond MP Huang H O’Brien LM Smith YR . Sleep habits of women with infertility. J Clin Endocrinol Metab. (2021) 106:e4414–26. doi: 10.1210/clinem/dgab474 34180998 PMC8530742

[14] Willis SK Hatch EE Wesselink AK Rothman KJ Mikkelsen EM Wise LA . Female sleep patterns, shift work, and fecundability in a North American preconception cohort study. Fertil Steril. (2019) 111:1201–1210.e1. doi: 10.1016/j.fertnstert.2019.01.037 30987736 PMC6546548

[15] Pavone M Hirshfeld-Cytron J Lawson A Smith K Klock S . Sleep distubances high in patients seeking fertility preservation. Fertil Steril. (2013) 100:S168. doi: 10.1016/j.fertnstert.2013.07.1463 25154674 PMC4253550

[16] Goldstein CA Lanham MS Smith YR O'Brien LM . Sleep in women undergoing *in vitro* fertilization: a pilot study. Sleep Med. (2017) 32:105–13. doi: 10.1093/med/9780197640739.003.0001 28366321 PMC5380145

[17] Yanık F Alus Tokat M . O-167 Sleep quality and affecting factors in women undergoing *in vitro* fertilization treatment. Hum Reprod. (2021) 36. doi: 10.1093/humrep/deab127.035

[18] Yao QY Yuan XQ Liu C Du YY Yao YC Wu LJ . Associations of sleep characteristics with outcomes of IVF/ICSI treatment: a prospective cohort study. Hum Reprod. (2022) 37:1297–310. doi: 10.1093/humrep/deac040 35259255

[19] Lin Y Chen Y Lin Y Xin S Ren A Zhou X . Association between sleep quality and ovarian reserve in women of reproductive age: a cross-sectional study. Fertil Steril. (2025) 123:520–8. doi: 10.1016/j.fertnstert.2024.09.018 39265649

[20] Xie Y Peng R Xiao L Li S Li X . Sleep quality during pregnancy following assisted reproductive technology and natural conceiving: a prospective birth cohort study. Front Endocrinol (Lausanne). (2024) 15:1497722. doi: 10.3389/fendo.2024.1497722 39911239 PMC11794090

[21] Wang SF Seifer DB . Assessment of a decade of change in U.S. assisted reproductive technology cumulative live-birth rates: 2004-2009 compared with 2014-2020. Obstet Gynecol. (2024) 143:839–48. doi: 10.1097/aog.0000000000005598 38696814

[22] Buysse DJ Reynolds CF Monk TH Berman SR Kupfer DJ . The Pittsburgh Sleep Quality Index: a new instrument for psychiatric practice and research. Psychiatry Res. (1989) 28:193–213. doi: 10.1016/0165-1781(89)90047-4 2748771

[23] Zhu Q Li Y Ma J Ma H Liang X . Potential factors result in diminished ovarian reserve: a comprehensive review. J Ovarian Res. (2023) 16:208. doi: 10.1186/s13048-023-01296-x 37880734 PMC10598941

[24] Ferraretti AP La Marca A Fauser BC Tarlatzis B Nargund G Gianaroli L . ESHRE consensus on the definition of 'poor response' to ovarian stimulation for *in vitro* fertilization: the Bologna criteria. Hum Reprod. (2011) 26:1616–24. doi: 10.1093/humrep/der092 21505041

[25] Hu S Xu B Jin L . Perinatal outcome in young patients with diminished ovarian reserve undergoing assisted reproductive technology. Fertil Steril. (2020) 114:118–124.e1. doi: 10.1016/j.fertnstert.2020.02.112 32622406

[26] Kim T Nam GE Han B Cho SJ Kim J Eum DH . Associations of mental health and sleep duration with menstrual cycle irregularity: a population-based study. Arch Womens Ment Health. (2018) 21:619–26. doi: 10.1007/s00737-018-0872-8 29909508

[27] Xing X Xue P Li SX Zhou J Tang X . Sleep disturbance is associated with an increased risk of menstrual problems in female Chinese university students. Sleep Breath. (2020) 24:1719–27. doi: 10.1007/s11325-020-02105-1 32445135

[28] Michels KA Mendola P Schliep KC Yeung EH Ye A Dunietz GL . The influences of sleep duration, chronotype, and nightwork on the ovarian cycle. Chronobiol Int. (2020) 37:260–71. doi: 10.1080/07420528.2019.1694938 31778080 PMC7054152

[29] Knight AK Hipp HS Abhari S Gerkowicz SA Katler QS McKenzie LJ . Markers of ovarian reserve are associated with reproductive age acceleration in granulosa cells from IVF patients. Hum Reprod. (2022) 37:2438–45. doi: 10.1093/humrep/deac178 35944168 PMC9527469

[30] Bachar G Gutzeit O Nebenzahl-Sharon K Weiner Z Beloosesky R Fainaru O . P-605 Hypoxia leads to diminished ovarian reserve in an age dependent manner. Hum Reprod. (2023) 38. doi: 10.21203/rs.3.rs-2801535/v1 38569488

[31] Lu X Liu Y Xu J Cao X Zhang D Liu M . Mitochondrial dysfunction in cumulus cells is related to decreased reproductive capacity in advanced-age women. Fertil Steril. (2022) 118:393–404. doi: 10.1016/j.fertnstert.2022.04.019 35637023

[32] Valsamakis G Chrousos G Mastorakos G . Stress, female reproduction and pregnancy. Psychoneuroendocrinology. (2019) 100:48–57. doi: 10.1016/j.psyneuen.2018.09.031 30291988

[33] Bala R Singh V Rajender S Singh K . Environment, lifestyle, and female infertility. Reprod Sci. (2021) 28:617–38. doi: 10.1007/s43032-020-00279-3 32748224

[34] Hua Z Ma D Xia X . Emotional dysregulation and time structure mediate the link between perceived stress and insomnia among unemployed young people in China: a cross-sectional study. Int J Environ Res Public Health. (2022) 19:11883. doi: 10.3390/ijerph191911883 36231183 PMC9564838

[35] Hughes A McMunn A Bartley M Kumari M . Elevated inflammatory biomarkers during unemployment: modification by age and country in the UK. J Epidemiol Community Health. (2015) 69:673–9. doi: 10.1136/jech-2014-204404 25700535 PMC4483793

[36] Barbaglia MG ten Have M Dorsselaer S Alonso J de Graaf R . Negative socioeconomic changes and mental disorders: a longitudinal study. J Epidemiol Community Health. (2015) 69:55–62. doi: 10.1136/jech-2014-204184 25205161

[37] Pal L Bevilacqua K Zeitlian G Shu J Santoro N . Implications of diminished ovarian reserve (DOR) extend well beyond reproductive concerns. Menopause. (2008) 15:1086–94. doi: 10.1097/gme.0b013e3181728467 18791485

[38] McAlpine CS Swirski FK . Circadian influence on metabolism and inflammation in atherosclerosis. Circ Res. (2016) 119:131–41. doi: 10.1161/circresaha.116.308034 27340272 PMC4922503

[39] Li Y Pei T Zhu H Wang R Wu L Huang X . Melatonin alleviates circadian rhythm disruption-induced enhanced luteinizing hormone pulse frequency and ovarian dysfunction. J Pineal Res. (2025) 77:e70026. doi: 10.1111/jpi.70026 39757996

[40] Sowers MF Zheng H Kravitz HM Matthews K Bromberger JT Gold EB . Sex steroid hormone profiles are related to sleep measures from polysomnography and the Pittsburgh Sleep Quality Index. Sleep. (2008) 31:1339–49. doi: 10.1143/JJAP.38.4333 PMC2572739 18853931

[41] Gong M Gao Y Wang Z Lu F Dong H . The impact of chronic insomnia disorder on menstruation and ovarian reserve in childbearing-age women: a cross-sectional study. Clin Exp Reprod Med. (2024) 51:142–50. doi: 10.5653/cerm.2023.06513 38812244 PMC11140260

