# Supplementary material for: Association between sleep and ovarian reserve in women undergoing IVF/ICSI: a Mendelian randomization and cross-sectional study
Source: Front Endocrinol (Lausanne). 2026 Sep 22;17:1970492. doi: 10.3389/fendo.2026.1970492 (PMC13638634; doi:10.3389/fendo.2026.1970492)
Supplement: Supplementary file 1 [file Table1.docx]

**Supplemental Table 1**

| **Variable assignment** | |
| --- | --- |
| **Variables** | **Assignment** |
| Educational level | Middle school and below (=0), High school (=1), College and above (=2) |
| Employed | No (=0), Yes (=1) |
| Smoking | Never (=0), ever (=1) |
| Alcohol drinking | No (=0), Yes (=1) |
| Hypnotic medications | No (=0), Yes (=1) |
| Salpingectomy history | No (=0), Yes (=1) |
| Dysmenorrhea | No (=0), Yes (=1) |
| Induced abortion | <3 (=0), ≥3 (=1) |
| Infertility | Primary infertility (=0), Secondary infertility (=1) |
| Daytime dysfunction | No (=0), Yes (=1) |
| Exercise | Hardly (=0), Occasionally (=1), Sometimes (=2), Frequently (=3) |
| Nocturnal sleep duration | ≥7h (=0), 6-7h (=1), <6h (=2) |
| Subjective sleep quality | Good (=0), Poor (=1) |
| Trouble falling asleep | 0 times/week (=0), 1 times/week (=1), 2 times/week (=2), 3 times/week (=3) |
| Sleep disturbance | No (=0), Yes (=1) |
| Night shift | No (=0), Occasionally (=1), Sometimes (=2), Frequently (=3) |
